# RAGE (Receptor for Advanced Glycation Endproducts), RAGE Ligands, and their role in Cancer and Inflammation

**DOI:** 10.1186/1479-5876-7-17

**Published:** 2009-03-17

**Authors:** Louis J Sparvero, Denise Asafu-Adjei, Rui Kang, Daolin Tang, Neilay Amin, Jaehyun Im, Ronnye Rutledge, Brenda Lin, Andrew A Amoscato, Herbert J Zeh, Michael T Lotze

**Affiliations:** 1 Department of Surgery, University of Pittsburgh Cancer Institute, Pittsburgh, USA; 2 Department of Biological Sciences, Carnegie Mellon University, Pittsburgh, USA; 3 Departments of Surgery and Bioengineering, University of Pittsburgh Cancer Institute, Pittsburgh, USA; 4 University of Pennsylvania, Philadelphia, USA; 5 Harvard University, Cambridge, USA; 6 Departments of Surgery, Bioengineering, and Pathology, University of Pittsburgh Cancer Institute, Pittsburgh, USA

## Abstract

The Receptor for Advanced Glycation Endproducts [RAGE] is an evolutionarily recent member of the immunoglobulin super-family, encoded in the Class III region of the major histocompatability complex. RAGE is highly expressed only in the lung at readily measurable levels but increases quickly at sites of inflammation, largely on inflammatory and epithelial cells. It is found either as a membrane-bound or soluble protein that is markedly upregulated by stress in epithelial cells, thereby regulating their metabolism and enhancing their central barrier functionality. Activation and upregulation of RAGE by its ligands leads to enhanced survival. Perpetual signaling through RAGE-induced survival pathways in the setting of limited nutrients or oxygenation results in enhanced autophagy, diminished apoptosis, and (with ATP depletion) necrosis. This results in chronic inflammation and in many instances is the setting in which epithelial malignancies arise. RAGE and its isoforms sit in a pivotal role, regulating metabolism, inflammation, and epithelial survival in the setting of stress. Understanding the molecular structure and function of it and its ligands in the setting of inflammation is critically important in understanding the role of this receptor in tumor biology.

## Review

### Introduction

The Receptor for Advanced Glycation Endproducts [RAGE] is a member of the immunoglobulin superfamily, encoded in the Class III region of the major histocompatability complex [1-4]. This multiligand receptor has one V type domain, two C type domains, a transmembrane domain, and a cytoplasmic tail. The V domain has two N-glycosylation sites and is responsible for most (but not all) extracellular ligand binding [5]. The cytoplasmic tail is believed to be essential for intracellular signaling, possibly binding to diaphanous-1 to mediate cellular migration [6]. Originally advanced glycation endproducts (AGEs) were indeed thought to be its main activating ligands, but since then many other ligands of RAGE including damage-associated molecular patterns (DAMP's) have been identified [1,7,8]. RAGE is thus considered a pattern-recognition receptor (PRR), having a wide variety of ligands [9-11].

**Figure 1:**
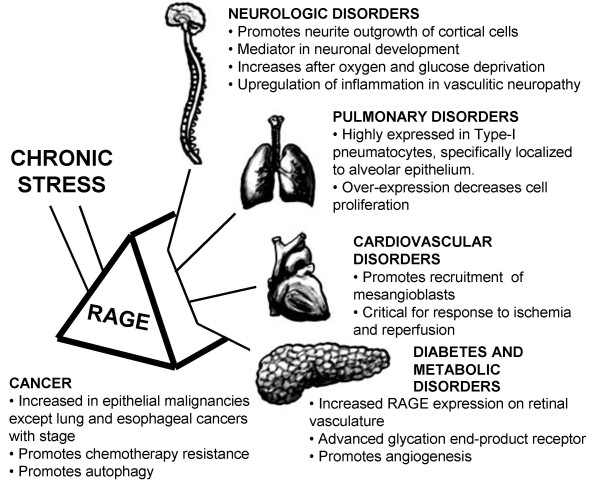
**RAGE is Central to Many Fundamental Biological Processes**. Focusing on RAGE allows us to view many aspects of disordered cell biology and associated chronic diseases. Chronic stress promotes a broad spectrum of maladies through RAGE expression and signaling, focusing the host inflammatory and reparative response.

RAGE is expressed as both full-length, membrane-bound forms (fl-RAGE or mRAGE, not to be confused with mouse RAGE) and various soluble forms lacking the transmembrane domain. Soluble RAGE is produced by both proteolytic cleavage of fl-RAGE and alternative mRNA splicing. The soluble isoforms include the extracellular domains but lack the transmembrane and cytoplasmic domains [12-15]. Soluble RAGE derived specifically from proteolytic cleavage is sRAGE, although this terminology is not consistent in the literature – sRAGE sometimes refers to soluble RAGE in general. RAGE is expressed at low levels in a wide range of differentiated adult cells in a regulated manner but in mature lung type-I pneumocytes it is expressed at substantially higher levels than in other resting cell types. It is highly expressed in readily detectable amounts in embryonic cells [16]. RAGE is also highly expressed and associated with many inflammation-related pathological states such as vascular disease, cancer, neurodegeneration and diabetes (Figure 1) [17,18]. The exceptions are lung tumors and idiopathic pulmonary fibrosis, in which RAGE expression decreases from a higher level in healthy tissue [19,20].

### RAGE and Soluble RAGE

Human RAGE mRNA undergoes alternative splicing, much as with other proteins located within the MHC-III locus on chromosome 6. A soluble form with a novel C-terminus is detected at the protein level, named "Endogenous Secretory RAGE" (esRAGE or RAGE_v1) [21]. This form is detected by immunohistochemistry in a wide variety of human tissues that do not stain for noticeable amounts of fl-RAGE [22]. Over 20 different splice variants for human RAGE have been identified to date. Human RAGE splicing is very tissue dependant, with fl-RAGE mRNA most prevalent in lung and aortic smooth muscle cells while esRAGE mRNA is prevalent in endothelial cells. Many of the splice sequences are potential targets of the nonsense-mediated decay (NMD) pathway and thus are likely to be degraded before protein expression. Several more lack the signal sequence on exon1 and thus the expressed protein could be subject to premature degradation. The only human variants that have been detected at the protein level *in vivo *is are fl-RAGE, sRAGE, and esRAGE [17,22].

Human fl-RAGE is also subject to proteolytic cleavage by the membrane metalloproteinase ADAM10, releasing the extracellular domain as a soluble isoform [12-14]. Antibodies raised to the novel C-terminus of esRAGE do not recognize the isoform resulting from proteolytic cleavage. In serum the predominant species is the proteolytic cleavage and not mRNA splicing isoform [12]. Enhancement of proteolytic cleavage will increase soluble RAGE levels, while inhibition will increase fl-RAGE levels. This cleavage process is modulated by Ca++ levels, and following proteolytic cleavage the remaining membrane-bound C-terminal fragment is subject to further degradation by γ-secretase [13,14]. Cleavage of the C-terminal fragment by γ-secretase will release a RAGE intercellular domain (RICD) into the cytosolic/nuclear space. Even though RICD has not yet been detected and is presumably degraded quickly, overexpression of a recombinant form of RICD will increase apoptosis as measured by TUNEL assay, indicating RAGE processing has another intercellular role [14].

Murine fl-RAGE mRNA also undergoes alternative splicing, and some of the splice products are orthologs of esRAGE [23]. To date over 17 different mRNA splices have been detected. As with human splice variants, mouse splice variants are expressed in a tissue-dependant fashion and many are targets of NMD. Several common splice patterns exist when comparing human and mouse RAGE, although variants that would give rise to a soluble isoform are much rarer in mice [15].

Recombinant RAGE has been cloned into a variety of expression vectors, and native soluble RAGE has been purified from murine, bovine, and human lung [24-28]. A recombinant soluble isoform takes on a dominant-negative phenotype and blocks signaling. Soluble RAGE can act as an extracellular "decoy receptor", antagonizing fl-RAGE and other receptors by binding DAMPs and other ligands and inhibiting leukocyte recruitment in a variety of acute and chronic inflammatory conditions [4]. Both esRAGE and sRAGE act as decoy receptors for the ligand HMGB1 [12]. However soluble RAGE has functions other than just blocking fl-RAGE function, and exerts pro-inflammatory properties through interaction with Mac-1 [10,29]. Thus although soluble RAGE has protective properties in the setting of chronic inflammation, it might be better described as a biomarker of chronic inflammation [30,12]. Information on long-term effects of treatment with exogenous soluble RAGE is still not available, and it has yet to be shown that plasma levels of soluble RAGE are sufficient to effectively act as a decoy receptor *in vivo *[18].

The two different properties of soluble RAGE (decoy receptor and pro-inflammatory) and the different pathways associated with its production might explain why there are both positive and negative correlations between its levels in human serum and disease. Total soluble RAGE in serum is significantly lower in non-diabetic men with coronary artery disease than those without [31]. As assessed by delayed-type hypersensitivity and inflammatory colitis, soluble RAGE suppressed inflammation In IL-10 deficient mice, reduced activation of NFκB, and reduced expression of inflammatory cytokines [32,33]. RAGE knockout mice have limited ability to sustain inflammation and impaired tumor elaboration and growth. Thus, RAGE drives and promotes inflammatory responses during tumor growth at multiple stages and has a central role in chronic inflammation and cancer [34].

Lower levels of soluble RAGE levels are found in Amyotrophic Lateral Sclerosis (ALS), and lower esRAGE levels predict cardiovascular mortality in patients with end-stage renal disease [35,36]. In patients with type 2 diabetes higher soluble RAGE levels positively correlate with other inflammatory markers such as MCP-1, TNF-α, AGEs, and sVCAM-1 [37,38]. Total soluble RAGE but not esRAGE correlates with albuminuria in type 2 diabetes [39]. Interestingly, although changes in human serum levels of soluble RAGE correlate very well with progression of inflammation-related pathologies, in mouse serum soluble RAGE is undetectable [18]. This contrasts the importance of splicing and proteolytic cleavage forms soluble RAGE in mice and humans [15]. One caution is that although ELISA-based assays of soluble RAGE in serum show high precision and reproducibility, the levels show high variation (500–3500 ng/L P < 0.05) among otherwise healthy donors [40]. Soluble RAGE levels correlate with AGE levels even in non-diabetic subjects [41]. Thus, although one measurement of soluble RAGE may not be sufficient to predict a pathological state, changes in levels over time could be predictive of the development of a disease.

### RAGE Signaling Perpetuates the Immune and Inflammatory Response

A recent review extensively covers the role of RAGE signaling in diabetes and the immune response [18]. Activation of multiple intracellular signaling molecules, including the transcription factor NF-κB, MAP kinases, and adhesion molecules are noted following activation of RAGE. The recruitment of such molecules and activation of signaling pathways vary with individual RAGE ligands. For example, HMGB1, S100B, Mac-1, and S100A6 activate RAGE through distinct signal transduction pathways [42,43]. Ann Marie Schmidt posited a "two-hit" model for vascular perturbation mediated by RAGE and its ligands [9]. This "two-hit" model hypothesizes that the first "hit" is increased expression of RAGE and its ligands expressed within the vasculature. The second "hit" is the presence of various forms of stress (e.g. ischemic stress, immune/inflammatory stimuli, physical stress, or modified lipoproteins), leading to exaggerated cellular response promoting development of vascular lesions. Most importantly, engagement of RAGE perpetuates NF-kB activation by de novo synthesis of NF-kBp65, thus producing a constantly growing pool of this pro-inflammatory transcription factor [44]. RAGE is associated with amplified host responses in several pathological conditions, including diabetes, chronic inflammation, tumors, and neurodegenerative disorders [18]. We would similarly posit that during periods of epithelial barrier disruption that both signal 1, a growth factor stimulus, and signal 2, various forms of stress, in conjunction with RAGE and RAGE ligands helps mediate this effect.

### RAGE Ligands

RAGE ligands fall into several distinct families. They include the High Mobility Group family proteins including the prototypic HMGB1/amphoterin, members of the S100/calgranulin protein family, matrix proteins such as Collagen I and IV, Aβ peptide, and some advanced glycation endproducts such as carboxymethyllysine (CML-AGE) [4,6,16,45]. Not all members of these families have been identified as RAGE ligands, and many RAGE ligands have a variety of RAGE-independent effects [46]. AGE molecules are prevalent in pathological conditions marked by oxidative stress, generation of methoxyl species, and increases in blood sugar, as found in type 2 diabetes mellitus [6,27]. The S100/calgranulin family consists of closely related calcium-binding polypeptides which act as proinflammatory extracellular cytokines.

Ligand accumulation and engagement in turn upregulates RAGE expression [2]. It is not known why some ligands (such as HMGB1, some S100's, and CML-AGE) cause strong pro-inflammatory signaling through RAGE, while similar molecules (such as pentosidine-AGE and pyrraline-AGE) seem to have much less or no signaling. The most commonly accepted hypothesis to reconcile these differences involves ligand oligomerization. Of the identified RAGE ligands, those that oligomerize activate RAGE more strongly [3]. Oligomers of ligands could potentially recruit several RAGE receptors as well as Toll-like receptors [TLRs] at the cell surface or at intracellular vesicles and induce their clustering on the cell surface. For example, S100 dimers and higher-order multimers bind several receptors including TLR4, and clustering of RAGE could promote a similarly strong response [47]. Recent studies show that AGEs and certain S100 multimers will cluster RAGE in this manner [11,48,49]. However this does not completely explain why some ligands will activate RAGE strongly while structurally similar ones do not seem to activate it at all [50].

### Overview of HMGB1 and the HMG Protein Family

HMG (High Mobility Group) proteins are very basic, nuclear, non-histone chromosomal proteins of which HMGB1 is the only member that has been shown to activate RAGE. The HMG proteins are not to be confused with the unrelated compound in the mevalonate pathway "HMG-CoA" (3-hydroxy-3-methylglutaryl coenzyme A) and "HMG-CoA reductase inhibitors" (statins) [51]. The HMG proteins were first identified in calf thymus in 1973 and named for their high mobility in protein separation gels [52]. Typically they have a high percentage of charged amino acids and are less than 30 kDa in mass. HMG proteins are expressed in nearly all cell types, relatively abundant in embryonic tissue, and bind to DNA in a content-dependant but sequence-independent fashion [53]. They are important in chromatin remodeling and have many other functions. Mouse knockout data shows that the loss of any one of the HMG proteins will result in detectable deleterious phenotypic changes. Of those, the HMGB1 (-/-) mice die of hypoglycemia within 24 hours of birth [54,55]. Extended back-crossing of the knockout allele into various murine strains have revealed an even more profound phenotype with mice dying by E15 of development [Marco Bianchi, personal communication]. The homology between mouse and human HMGB1 is extraordinary with only two amino acid differences observed. Similar profound homology exists throughout vertebrate species with 85% homology with zebrafish.

There are three sub-classifications of HMG proteins: HMGA, HMGB, and HMGN (Table 1). There is also a similar set known as HMG-motif proteins. The HMG-motif proteins differ in that they are cell-type specific, and bind DNA in a sequence-specific fashion. HMGA proteins (formerly HMGI/Y) are distinguished from other HMG proteins by having three AT-hook sequences (which bind to AT-rich DNA sequences) [56,57]. They also have a somewhat acidic C-terminal tail, although the recently discovered HMGA1c has no acidic tail and only two AT-hooks. HMGN proteins (formerly HMG14 and HMG17) have nucleosomal binding domains. HMGB proteins (formerly HMG1 through HMG4) are distinguished by having two DNA-binding boxes that have a high affinity for CpG DNA, apoptotic nuclei, and highly bent structures such as four-way Holliday junctions and platinated/platinum-modified DNA. The HMGB proteins have a long C-terminal acidic tail except for HMGB4, which recently has been detected at the protein level in the testis where it acts as a transcriptional repressor [58]. The HMGB acidic tail consists of at least 20 consecutive aspartic and glutamic acid residues. A C-terminal acidic tail of this length and composition is rarely seen in Nature, although a few other autophagy and apoptosis-related proteins such as parathymosin have a long internal stretch of acidic peptides [59-61].

**Table 1:** MG Proteins in Cancer and Normal Tissues

**Name** **(alt. name)**	**Chromosome**	**Post-translational modifications**	**Sub-cellular localization**	**Normal tissue expression**	**Expression in cancer**
**HMGA1a **(HMG-I, HMG-I/Y), **HMGA1b **(HMG-Y), **HMGA1c **(HMG-I/R)	6p21	Highly modified with numerous sites of phosphorylation, acetylation and/or methylation. Possibly SUMOylated and ADP-ribosylated.	Nucleus but has role in shuttling HIPK2 (homeodomain-interacting protein kinase 2) to the cytosol	Abundantly expressed in undifferentiated and proliferating embryonic cells but usually undetectable in adult tissue	Overexpressed in malignant epithelial tumors and leukemia

**HMGA2 **(HMGI-C, HMGIC)	12q14-15	Phosphorylated	Nucleus – the second AT-hook is necessary and sufficient for nuclear localization	See HMGA1's	Invasive front of carcinomas. A splice variant without the acidic tail is found in some benign tumors.

**HMGB1 **(HMG1, Amphoterin)	13q12	Acetylated, methylated, phosphorylated, and/or ADP-ribosylated when actively secreted. An acidic tail-deleted isoform has been purified from calf thymus	Often nuclear but translocates to the cytosol and is actively secreted and passively released	Abundantly expressed in all tissues except neurons. Highest levels in thymus, liver and pancreas.	See Table 2

**HMGB2 **(HMG2)	4q31	Phosphorylated on up to three residues	see HMGB1	Thymus and testes	Squamous cell carcinoma of the skin, ovarian cancer

**HMGB3 **(HMG-4, HMG-2a)	Xq28			Lymphoid organs. mRNA detected in embryos and mouse bone marrow	mRNA detected in small cell and non-small cell lung carcinomas (SCLC, NSCLC)

**HMGN1 **(HMG14)	21q22.3	Acetylated, highly phosphorylated,	nucleus	Weakly expressed in most tissues	

**HMGN2 **(HMG17)	1p36.1-1p35	Acetylated	nucleus	Weakly expressed in most tissues, but strong in thymus, bone marrow, thyroid and pituitary gland	

**HMGN3 **(TRIP-7)	6q14.1		nucleus	Abundantly expressed in kidney, skeletal muscle and heart. Low levels found in lung, liver and pancreas	

**HMGN4 **(HMG17, L3 NHC)	6p21.3	Highly phosphorylated	nucleus	Weakly expressed in all tissues	

Of the HMG proteins, HMGB1 has an additional cytosolic and extracellular role as a protein promoting autophagy and as a leaderless cytokine, respectively [62]. Macrophages, NK cells and mature DCs actively secrete HMGB1, and necrotic cells passively secrete it. HMGB1 has also been detected in the cytosol, depending on the cell type, where it has a major positive role in regulating autophagy [63]. Although HMGA1 has a role in the export of HIPK2 (Homeodomain-interacting protein kinase 2, a proapoptotic activator of p53) from the nucleus to the cytoplasm [64], the HMG proteins other than HMGB1 are very seldom detected outside the nucleus. This is likely explains why HMGB1 is the only member of the family that activates RAGE [65]. Since HMGB1 translocates between the nucleus and cytosol, there is a possibility that it could bind to soluble RAGE in the cytosol and thereby play a role in regulating its activity.

### Biochemistry of HMGB1

HMGB1 is a highly conserved protein consisting of 215 amino acids. It is expressed in almost all mammalian cells. Human HMGB1 shares an 80% similarity with HMGB2 and HMGB3 [55]. It has two lysine-rich DNA binding boxes (A- and B-) separated by a short linker. The boxes are separated from the C-terminal acidic tail by another linker sequence ending in four consecutive lysines. An isoform believed to result from cleavage of the acidic tail has been detected *in vivo *[66]. HMGB1 has three cysteines, of which the first two vicinal cysteines (Cys 23 and 45, based on Met1 as the initial Met in the immature protein) can form an internal disulfide bond within the A-box. The A-box and the oxidation state of these two cysteines play an important role in the ability of HMGB1 to bind substrates. Oxidation of these two cysteines will also reduce the affinity of HMGB1 for CpG-DNA [67,68]. Addition of recombinant A-box antagonizes HMGB1's ability to bind other substrates [67,69]. It remains to be determined if the action of the A-box is the result of competitive inhibition by binding to other substrates or interfering with the ability of the B-box to bind substrates. The two boxes acting in concert will recognize bent DNA [70]. The third cysteine (Cys106, in the B-box) often remains reduced and is important for nuclear translocation [68]. The region around this cysteine is the minimal area with cytokine activity [65]. HMGB1 undergoes significant post-translational modification, including acetylation of some lysines, affecting its ability to shuttle between the nucleus and cytosol [71,72]. DNA-binding and post-translational modification accessibility can be modulated by interactions of the acidic tail with the basic B-box [73-75]. HMGB1 signals through TLR2, TLR4, and TLR9 in addition to RAGE [76,77]. It also binds to thrombomodulin and syndecan through interactions with the B-box [78].

### Evolution of HMGB1

HMG proteins can be found in the simplest multi-cellular organisms [79]. The two DNA boxes resulted from the fusion of two individual one-box genes [80]. The two-box structure makes it particularly avid specific for bent DNA, and is highly conserved among many organisms [81,82]. This similarity makes generation of HMGB1-specific antibodies a challenge. Antibody cross-reactivity could result from the strong similarity of HMGB1 across individual species, HMGB1 to other HMGB proteins, and even HMGB1 to H1 histones (Sparvero, Lotze, and Amoscato, unpublished data). The possibility of misidentification of HMGB1 must be ruled out carefully in any study. One way to distinguish the HMGB proteins from each other is by the length of the acidic tail (30, 22, and 20 consecutive acidic residues for HMGB1, 2, and 3 respectively, while HMGB4 has none). The acid tails are preceded by a proximal tryptic cleavage site, and they all have slightly different compositions. This makes mass spectrometry in conjunction with tryptic digestion an attractive means of identification.

### Normal/healthy levels of HMGB1

Relative expression of HMGB1 varies widely depending on tissue condition and type. Undifferentiated and inflamed tissues tend to have greater HMGB1 expression than their counterparts. Spleen, thymus and testes have relatively large amounts of HMGB1 when compared to the liver. Subcellular location varies, with liver HMGB1 tending to be found in the cytosol rather than the nucleus [55,83]. HMGB1 is present in some cells at levels exceeded only by actin and estimated to be as much as 1 × 10^6 ^molecules per cell, or one-tenth as abundant as the total core histones. But this number should be regarded with some caution since it includes transformed cell lines and does not define the levels of HMGB1 abundance *in vivo *in most cellular lineages [55]. The levels of serum HMGB1 (as determined by Western Blot) have been reported with wide ranges: 7.0 ± 5.9 ng/mL in healthy patients, 39.8 ± 10.5 ng/mL in cirrhotic liver and 84.2 ± 50.4 ng/mL in hepatocellular carcinoma [84]. For comparison, human total serum protein levels vary from about 45–75 mg/mL, and total cytosolic protein levels are about 300 mg/mL [85,86]. This puts serum HMGB1 in the low part-per-million range by mass, making detection and separation from highly abundant serum proteins challenging.

### HMGB1 and RAGE in cancer and inflammation

HMGB1, along with RAGE, is upregulated in many tumor types (Table 2). HMGB1 is passively released from necrotic cells but not from most apoptotic cells. The reason for this is unknown, but has been hypothesized to be a result of either redox changes or under-acetylation of histones in apoptotic cells [87,88]. HMGB1(-/-) necrotic cells are severely hampered in their ability to induce inflammation. HMGB1 signaling, in part through RAGE, is associated with ERK1, ERK2, Jun-NH2-kinase (JNK), and p38 signaling. This results in expression of NFκB, adhesion molecules (ICAM, and VCAM, leading to macrophage and neutrophil recruitment), and production of several cytokines (TNFα, IL-1α, IL-6, IL-8, IL-12 MCP-1, PAI-1, and tPA) [89]. An emergent notion is that the molecule by itself has little inflammatory activity but acts together with other molecules such as IL-1, TLR2 ligands, LPS/TLR4 ligands, and DNA. HMGB1 signaling through TLR2 and TLR4 also results in expression of NFκB. This promotes inflammation through a positive feedback loop since NFκB increases expression of various receptors including RAGE and TLR2. LPS stimulation of macrophages will lead to early release of TNFα (within several hours) and later release of HMGB1 (after several hours and within a few days). Targeting HMGB1 with antibodies to prevent endotoxin lethality therefore becomes an attractive therapeutic possibility, since anti-HMGB1 is effective in mice even when given hours following LPS stimulation [90]. HMGB1 stimulation of endothelial cells and macrophages promotes TNFα secretion, which also in turn enhances HMGB1 secretion [91]. Another means to induce HMGB1 secretion is with oxidant stress [92]. The actively secreted form of HMGB1 is believed to be at least partially acetylated, although both actively and passively released HMGB1 will promote inflammation [71].

**Table 2:** HMGB1 and RAGE in Cancer and Inflammation

**Inflammatory state, disease or cancer**	**Effect of RAGE/HMGB1**
Colon cancer	Co-expression of RAGE and HMGB1 leads to enhanced migration and invasion by colon cancer cell lines. Increased RAGE expression in colon cancer has been associated with atypia, adenoma size, and metastasis to other organs. Stage I tumors have relatively low % of tumors expressing, Stage IV virtually universal expression

Prostate cancer	Co-expression of RAGE and HMGB1 has been found in a majority of metastatic cases, in tumor cells and associated stromal cells.

Pancreatic cancer	Enhanced expression of RAGE and HMGB1 in the setting of metastases.

Lung and esophageal cancers	Higher tumor stage is characterized by downregulation of RAGE.

Inflammatory Arthritis	HMGB1 is overexpressed. RAGE binding, as other receptors, results in: macrophage stimulation, induction of TNFα and IL-6, maturation of DCs, Th1 cell responses, stimulation of CD4+ and CD8+ cells, and amplification of response to local cytokines.

Sepsis	HMGB1 propagates inflammatory responses and is a significant RAGE ligand in the setting of sepsis and acute inflammation. HMGB1 is an apparent autocrine/paracrine regulator of monocyte invasion, involving RAGE mediated transmigration through the endothelium.

An early observation dating back to 1973 is that the HMG proteins aggregate with less basic proteins [52]. HMGB1 binds LPS and a variety of cytokines such as IL-1β. This results in increased interferon gamma (INFγ) production by PBMC (peripheral blood mononuclear cells) that is much greater than with just HMGB1 or cytokines alone. HMGB1 binding to RAGE is enhanced with CpG DNA. HMGB1's ability to activate RAGE may result more from its ability to form a complex with other pro-inflammatory molecules, with this complex subsequently activating RAGE [93]. Therefore any test of RAGE binding solely by HMGB1 will have to account for this, since contamination with even small amounts of LPS or CpG DNA will increase binding. Thrombomodulin competes with RAGE for HMGB1 *in vitro *and the resulting complex does not appear to bind RAGE, suggesting a possible approach to attenuate RAGE-HMGB1 signaling [78,94]. In fact binding to thrombomodulin can also lead to proteolytic cleavage of HMGB1 by thrombin, resulting in a less-active inflammatory product [94].

A peptide consisting of only residues 150–183 of HMGB1 (the end of the B-box and its linker to the acidic tail) exhibits RAGE binding and successfully competes with HMGB1 binding *in vitro *[95]. This sequence ias similar to the first 40 amino acids (the first EF-hand helix-loop-helix sequence) of several S100 proteins. An HMGB1 mutant in which amino acids 102–105 (FFLF, B-box middle) are replaced with two glycines induces significantly less TNFα release relative to full length HMGB1 in human monocyte cultures [96]. This mutant is also able to competitively inhibit HMGB1 simulation in a dose-dependent manner when both are added.

### Is HMGB1 the lone RAGE activator of the HMG family?

For all the reasons noted above, HMGB1 is the sole known HMG-box ligand of RAGE. None of the other nuclear HMG proteins have been shown to activate RAGE. The HMGB proteins can complex CpG DNA, and highly bent structures such as four-way Holliday junctions and platinated/platinum-modified DNA while other members cannot. Unlike other HMGB proteins, HMGB1 is abundantly expressed in nearly all tissues, and thus is readily available for translocation out of the nucleus to the cytosol for active and passive secretion. Although as a cautionary note, HMGB2 and HMGB3 are also upregulated in some cancers, and might play a role as RAGE activators in addition to HMGB1. The similarity of these proteins to HMGB1 suggests in various assays that they may be misidentified and included in the reported HMGB1 levels. The HMG and S100 family members each consist of similar proteins that have distinct and often unapparent RAGE-activating properties.

### S100 Proteins as RAGE ligands and their role in Inflammation

A recent review on S100 proteins has been published, and provides more extensive detail than given here [97]. We will focus on the critical elements necessary to consider their role in cancer and inflammation. S100 proteins are a family of over 20 proteins expressed in vertebrates exclusively and characterized by two calcium binding EF-hand motifs connected by a central hinge region [98]. Over forty years ago the first members were purified from bovine brain and given the name "S-100" for their solubility in 100% ammonium sulfate [99]. Many of the first identified S100 proteins were found to bind RAGE, and thus RAGE-binding was theorized to be a common property of all S100 proteins. However several of the more recently identified members of the family do not bind RAGE. The genes located on a cluster on human chromosome 1q21 are designated as the *s100a *sub-family and are numbered consecutively starting at *s100a1*. The S100 genes elsewhere are given a single letter, such as *s100b *[100]. In general, mouse and human S100 cDNA is 79.6–95% homologous although the mouse genome lacks the gene for S100A12/EN-RAGE [101]. Most S100 proteins exist as non-covalent homodimers within the cell [98]. Some form heterodimers with other S100 proteins – for example the S100A8/S100A9 heterodimer is actually the preferred form found within the cell. The two EF-hand Ca++ binding loops are each flanked by α-helices. The N-terminal loop is non-canonical, and has a much lower affinity for calcium than the C-terminal loop. Members of this family differ from each other mainly in the length and sequence of their hinge regions and the C-terminal extension region after the binding loops. Ca++ binding induces a large conformational change which exposes a hydrophobic binding domain (except for S100A10 which is locked in this conformation) [47]. This change in conformation allows an S100 dimer to bind two target proteins, and essentially form a bridge between as a heterotetramer [102]. The S100 proteins have been called "calcium sensors" or "calcium-regulated switches" as a result. Some S100 proteins also bind Zn++ or Cu++ with high affinity, and this might affect their ability to bind Ca++ [101].

S100 proteins have wildly varying expression patterns (Table 3). They are upregulated in many cancers, although S100A2, S100A9, and S100A11 have been reported to be tumor repressors [50]. S100 proteins and calgranulins are expressed in various cell types, including neutrophils, macrophages, lymphocytes, and dendritic cells [2]. Phagocyte specific, leaderless S100 proteins are actively secreted via an alternative pathway, bypassing the Golgi [103]. Several S100 proteins bind the tetramerization domain of p53, and some also bind the negative regulatory domain of p53. Binding of the tetramerization domain of p53 (thus controlling its oligomerization state) could be a property common to all S100 proteins but this has not been reported [104]. Their roles in regulating the counterbalance between autophagy and apoptosis have also not been reported.

**Table 3:** S100 Proteins in Cancer and Normal Tissues

**Name**	**Chrom**.	**RAGE binding**	**p53 binding**	**Normal tissue expression**	**Expression in cancer**	**Cancer notes**
**S100A1**	1q21	**Possibly, (antagonizes S100A4-RAGE interactions)**	Yes – TET and NRD	Highest in heart, also expressed in kidney, liver, skin, brain, lung, stomach, testis, muscle, small intestine, thymus and spleen	Renal carcinoma	

**S100A2**	1q21	**Not observed**	Yes – TET and NRD	Kerotinocytes, breast epithelial tissue, smooth muscle cells and liver	Thyroid, prostate, lung, oral, and breast carcinomas; melanoma	Mostly down-regulated but upregulated in some cancer types

**S100A3**	1q21	**Not observed**		Differentiating cuticular cells in the hair follicile		

**S100A4**	1q21	**Yes, coexpressed with RAGE in lung and breast cancer**		Chondrocytes, astrocytes, Schwann cells, and other neuronal cells	Thyroid, breast and colorectal carcinomas; melanoma; bladder and lung cancers	Overexpression is associated with metastases and poor prognosis

**S100A5**	1q21	**Not observed**		Limited areas of the brain	Astrocytic tumors	Overexpressed

**S100A6**	1q21	**Yes, coexpressed with RAGE in lung and breast cancer**	Yes – TET	Neurons of restricted regions of the brain	Breast cancer, colorectal carcinoma	Not found in healthy breast or colorectal

**S100A7/A7A**	1q21	**Yes, Zinc dependant activation**		Kerotinocytes, dermal smooth muscle cells	Breast carcinoma, bladder and skin cancers	Not expressed in non-cancer tissues except for skin

**S100A8/A9**	1q21	**Possibly (activates NF-kB in endothelial cells)**		Expressed and secreted by neutrophils	Breast and colorectal carcinomas, gastric cancer	Upregulated in premetastatic stage, then downregulated

**S100A9**	1q21	**See S100A8**		See S100A8	See S100A8	

**S100A10**	1q21	**Not observed**		Several tissues, highest in lung, kidney, and intestine		

**S100A11**	1q21	**Yes – inflammation induced chondrcyte hypertrophy**	Yes – TET	Keratinocytes	Colorectal, breast, and renal carcinomas; bladder, prostate, and gastric cancers	Decreased expression is an early event in bladder carcinoma, high expression is associated with better prognosis in bladder and renal cancer patients but worse prognosis in prostate and breast

**S100A12**	1q21	**Yes – Inflammatory processes (activates endothelial cells and leukocytes)**		Granulocytes, keratinocytes	Expressed in acute, chronic, and allergic inflammation	

**S100A13**	1q21	**Yes – stimulates its own uptake by cells**		Broadly expressed in endothelial cells, but not vascular smooth muscle cells		Upregulated in endometrial lesions

**S100A14**	1q21	**Not observed**		Broadly expressed in many tissues, but not detected in brain, skeletal muscle, spleen, peripheral blood leukocytes		Overexpressed in ovary, breast and uterus tumors, Down-regulated in kidney, rectum and colon tumors

**S100A15** **(name withdrawn, see S100A7)**						

**S100A16**	1q21	**Not observed**		Broadly expressed with highest levels esophagus, lowest in lung, brain, pancreas and skeletal muscle		Upregulated in lung, pancreas, bladder, thyroid and ovarian tumors

**S100B**	21q22	**Yes – RAGE -dependant, cytochrome C mediated activation of caspase-3**	Yes – TET and NRG	Astrocytes	Melanoma	Overexpressed in melanoma

**S100G**	Xp22	**Not observed**		Pancreas, intestine, mineralized tissues	Pancreatic cancer	Overexpressed >100-fold

**S100P**	4p16	**Yes – stimulates cell proliferation and survival**		Placenta	Prostate and gastric cancers	Overexpressed

**S100Z**	5q14	**Not observed**		Pancreas, lung, placenta, and spleen		Decreased expression in cancer

p53 binding domains: TET: Tetramerization, NRD: Negative regulatory domain

Individual S100 proteins are prevalent in a variety of inflammatory diseases, specifically S100A8/A9 (which possibly signals through RAGE in addition to other mechanisms), and S100A12 (which definitely signals through RAGE). These diseases include rheumatoid arthritis, juvenile idiopathic arthritis, systemic autoimmune disease and chronic inflammatory bowel disease. Blockade of the S100-RAGE interaction with soluble RAGE in mice reduced colonic inflammation in IL-10-deficient mice, inhibited arthritis development, and suppressed inflammatory cell infiltration [43,33,32,105]. Some S100 proteins have concentration-dependant roles in wound healing, neurite outgrowth, and tissue remodeling.

There are several important questions that need to be addressed when examining proposed S100-RAGE interactions: Does this interaction occur *in vivo *in addition to *in vitro*? Could the observed effects be explained by a RAGE-independent mechanism (or even in addition to a non-RAGE mechanism)? Is this interaction dependant on the oligomeric state of the S100 protein? (S100 oligomeric state is itself dependant on the concentration of Ca++ and other metal ions as well as the redox environment). One area that has not received much attention is the possibility of S100 binding to a soluble RAGE in the cytosol or nucleus (as opposed to extracellular soluble RAGE).

### S100 Proteins are not universal RAGE ligands

Several of the S100 family members are not RAGE ligands. Although there is no direct way to identify RAGE binding ability based on the amino acid sequences of the S100 proteins, conclusions can be drawn based on common biochemical properties of the known S100 non-ligands of RAGE: The first is that the non-ligands often exhibit strong binding to Zn++. The second is that their Ca++ binding is hindered or different in some ways from the S100 RAGE ligands. The third is that their oligomerization state is altered or non-existent.

### Non-ligands of RAGE: S100A2, A3, A5, A10, A14, A16, G, Z

S100A2 is a homodimer that can form tetramers upon Zn++ binding, and this Zn++ binding inhibits its ability to bind Ca++. Although two RAGE ligands (S100B and S100A12) also bind Zn++ very well, the effect on them is to increase their affinity for Ca++ [106,107]. The related S100A3 binds Ca++ poorly but Zn++ very strongly [101]. S100A5 is also a Zn++ binder, but it binds Ca++ with 20–100 fold greater affinity than other S100 proteins. It also can bind Cu++, which will hinder its ability to bind Ca++ [108]. S100A10 (or p11) is the only member of the S100 family that is Ca++ insensitive. It has amino acid alterations in the two Ca++ binding domains that lock the structure into an active state independently of calcium concentration [109]. It will form a heterotetramer with Annexin A2, and it has been called "Annexin A2 light chain" [110]. S100A14 has only 2 of the 6 conserved residues in the C-terminal EF-hand, and thus its ability to bind Ca++ is likely hindered [111]. S100A16 binds Ca++ poorly, with only one atom per monomer of protein. However upon addition of Zn++, higher aggregates form [112]. S100G was also known as Vitamin D-dependent calcium-binding protein, intestinal CABP, Calbindin-3, and Calbindin-D9k [113]. It is primarily a monomer in solution and upon Ca++ binding it does not exhibit the conformational changes that characterize many other S100 proteins [114]. S100Z is a 99-amino acid protein that binds S100P *in vitro*. It exists as a homodimer that binds Ca++ but its aggregation state is unaffected by Ca++ [115].

### Possible ligands of RAGE: S100A1, S100A8/9

S100A1 normally exists as a homodimer, and its mRNA is observed most prominently in the heart, with decreasing levels in kidney, liver, skin, brain, lung, stomach, testis, muscle, small intestine, thymus and spleen. S100A1 is present in the cytoplasm and nucleus – rat heart muscle cell line H9c2 is mostly nuclear, adult skeletal muscle mostly cytoplasmic. S100A1 is released into the blood during ischemic periods, and extracellular S100A1 inhibits apoptosis via ERK1/2 activation [101]. S100A1 binds to both the tetramerization and negative regulatory domains of p53 [104]. S100A1 interacts with S100A4 and they antagonize each other *in vitro *and *in vivo *[116]. There is still some debate if S100A1 binds to RAGE, although recent work with PET Imaging of Fluorine-18 labeled S100A1 administered to mice indicates that it co-localizes with RAGE [117].

In addition to forming homodimers, S100A8 and S100A9 can form heterodimers and heterotetramers with each other in a calcium and oxidation-dependant fashion [101]. S100A8 and S100A9 have not been directly shown to activate RAGE, but there is substantial functional evidence that many of their effects are blocked by RAGE suppression or silencing. S100A8/9 exerts a pro-apoptotic effect in high concentrations, but promotes cell growth at low concentrations [118]. The effects of N-carboxymethyl-lysine-modified S100A8/9 are ameliorated in RAGE knockout mice or by administration of soluble RAGE to wild-type mice [119]. S100A8/9 binds to heparan sulfate, proteoglycans, and carboxylated N-glycans [103]. A small (<2%) sub-population of RAGE expressed on colon tumor cells are modified to carboxylated N-glycans, and it is quite possible that this sub-population is activated by S100A8/9 [120]. S100A8 and S100A9 proteins are secreted in an energy-dependent fashion by phagocytes during inflammatory processes. They stimulate specific inflammatory patterns in endothelial cells, interacting with components of the cytoskeleton, including keratin filaments, microtubules, type III intermediate filaments, and F-actin [121]. In the presence of calcium, S100A8 and S100A9 form tetramers and bind directly to microtubules. S100A8/S100A9 also modulates tubulin-dependent cytoskeleton rearrangement during migration of phagocytes. S100A8 and S100A9 interact with both type III intermediate filaments and keratin filaments for the purpose of wound repair. Extracellularly, the S100A8/S100A9 complex displays cystostatic and antimicrobial activities and inhibits macrophage activation and immunoglobulin synthesis by lymphocytes [122]. The reduced but not the oxidized S100A8 homodimer is strongly chemotactic for leukocytes [121]. Upregulation of S100A8 and S100A9 in premetastatic lung tissue provide a niche for migration of tumor cells [123]. This upregulation can be induced by VEGF-A, TGFβ and TNFα secretion from distant tumors. Upregulation in lung VE-cadherin+ endothelial cells promotes recruitment and infiltration of Mac1+ myeloid cells, and thus provides a niche for migration of tumor cells. Blocking S100A8 and S100A9 expression in the premetastatic stage could prevent this permissive niche from being formed and thus inhibit the migration of tumor cells.

### Ligands of RAGE: S100A4, A6, A7/A7A/A15, A11, A12, A13, B, P

#### S100A4

S100A4 binds to RAGE, and has been implicated in upregulation of MMP-13 (Matrix Metalloproteinase 13) in osteoarthritis, which leads to tissue remodeling [124]. S100A4 is expressed in astrocytes, Schwann cells, and other neuronal cells in addition to chondrocytes [43]. S100A4 is upregulated after nerve tissue injury. Neurite outgrowth stimulated by S100A4 is observed for the protein in the oligomeric, not dimeric, state [121]. This protein also stimulates angiogenesis via the ERK1/2 signaling pathway. S100A4 binds to the tetramerization domain but not the negative regulatory domain of p53 [104].

#### S100A6

S100A6 is found primarily in the neurons of restricted regions of the brain [42]. S100A6 is also found in the extracellular medium of breast cancer cells. S100A6 binds to the tetramerization domain of p53 [104]. S100A6 bound significantly to the C2 domain of RAGE, as opposed to the V and/or C1 domains to which most other ligands bind and thus suggests that it might have a discordant function from other RAGE ligands. S100A6 triggers the JNK pathway and subsequently the Caspase 3/7 pathway, resulting in apoptosis [125].

#### S100A7/S100A7A/S100A15

S100A7, also called Psoriasin 1, is part of a sub-family of several proteins [101]. The highly homologous S100A7A, a member of this sub-family, was formerly known as S100A15 but this name has been withdrawn [113]. S100A7 and S100A7A are functionally distinct despite the high sequence similarity [126]. S100A7 is highly expressed in epidermal hyperproliferative disease and recruits CD4+ lymphocytes and neutrophils [102]. Although several S100 proteins are upregulated in various forms of breast cancer, S100A7 is strongly up-regulated only in ductal carcinoma *in situ *[127]. There are high levels of monomeric and covalently crosslinked high molecular weight S100A7 in human wound exudate and granulation tissue. Immunohistological studies suggest that S100A7 is produced by keratinocytes surrounding the wound and is released into the wound exudate. S100A7 exerts antibacterial activity, and the central region including only amino acids 35–80 is sufficient to mediate this activity [128]. Although both S100A7 and S100A7A are expressed in keratinocytes, S100A7A is also expressed in melanocytes and Langerhans cells of the epidermis, and dermal smooth muscle endothelial cells [126]. Binding, signaling, and chemotaxis of S100A7 are dependent on Zn++ and RAGE in vitro, while S100A7A seems to signal through a RAGE-independent pathway. S100A7 and S100A7A exert a synergistic effect, promoting inflammation *in vivo *[126].

#### S100A11

S100A11 is overexpressed in many cancers [129]. It is homodimeric and interacts in a Ca++ dependant fashion with annexin I [130]. It is a key mediator of growth of human epidermal keratinocytes triggered by high Ca++ or TGFβ [129]. Under these conditions S100A11 is phosphorylated and transported to the nucleus by nucleolin. S100A11 binds to the tetramerization domain (but not the negative regulatory domain) of p53 [104]. Extracellular S100A11 is dimerized by transglutaminase 2, and this covalent homodimer acquires the capacity to signal through the p38 MAPK pathway, accelerate chondrocyte hypertrophy and matrix catabolism, and thereby couples inflammation with chondrocyte activation to promote osteoarthritis progression [131].

#### S100A12/EN-RAGE

S100A12 (EN-RAGE) is primarily expressed in granulocytes, but also in found in keratinocytes and psoriatic lesions. S100A12 represents about 5% of the total cytosolic protein in resting neutrophils. It is expressed in acute, chronic, and allergic inflammation. It interacts with RAGE in a Ca++ dependent manner, but also binds Cu++. There is no *s100a12 *gene in mice, although S100A8 seems to be a functional homologue [132,133]. S100A12 is up regulated in psoriasis and melanoma [101]. It binds to the RAGE C1 and C2 domains instead of the V domain [49]. It can also bind to RAGE expressed on endothelial cells, signaling through the NF-κB and MAPK pathways. S100A12 shares sequence homology with the putative RAGE-binding domain of HMGB1 (residues 153–180). Secreted S100A12 binds to RAGE and enhances expression of intercellular adhesion molecule-I (ICAM-1), vascular cell adhesion molecule-I (VCAM-1), NF-κB, and tumor necrosis factor (TNF)-α [43]. S100A12 is a chemoattractant for monocytes and mast cells, although only the hinge region seems important for the latter [134]. Since mast cells do not express RAGE protein or mRNA, their activation by S100A12 occurs in a RAGE-independent fashion. S100A12 exists as a homodimer under low Ca++ conditions, but will form hexamer aggregates (three dimers) at millimolar concentrations of Ca++ [135]. S100A12, in addition to S100A13, binds to the anti-allergic drugs cromolyn, tranilast, and amlexanox in a Ca++ dependant manner. This suggests that S100A12 and S100A13 might be involved in degranulation of mast cells in a RAGE-independent manner [136].

#### S100A13

S100A13 has a very broad expression pattern, in contrast to the other S100 proteins. S100A13 is expressed in endothelial cells, but not vascular smooth muscle cells. It is upregulated in extra-uterine endometriosis lesions when compared to normal tissues, and may have a role in vascularization [137]. Its affinity for Ca++ is low, but Ca++ binding leads to a conformational change exposing a novel Cu++ binding site [138]. Upon Cu++ binding, it regulates the stress-dependant release of FGF-1 and plays a role in angiogenesis in high-grade astrocytic gliomas [139]. S100A13 in addition to S100A12 may be involved in the degranulation of mast cells [136].

#### S100B

S100B is expressed primarily in the astrocytes of the human cortex and melanocytes as well as myeloid dendritic cells [42]. S100B, along with S100A1 and S100A6, are the most abundant S100 proteins in the brain of several species including mice and rats. Elevated levels of S100B have been found in patients following brain trauma, ischemia/infarction, Alzheimer's disease, and Down's syndrome [42]. S100B is used as a marker of glial cell activation and death [140]. It is believed to exist as a mixture of covalent and non-covalent dimers in the brain since ELISA assays done under non-oxidizing conditions will underestimate the amount of S100B [141,142]. In this regard, covalent S100B dimers can be used as a marker of oxidative stress [142]. S100B binds to both the tetramerization domain and the negative regulatory domain of p53 [104]. S100B also inhibits microtubulin and type III intermediate filament assemblies. S100B binds both the variable (V) and constant (C1) regions of RAGE, and oligomers of S100B bind RAGE more strongly [42,48]. At equivalent concentrations, S100B increases cell survival while S100A6 induces apoptosis via RAGE interactions, dependant on generation of reactive oxygen species (ROS). Upon binding to RAGE and activating intracellular ROS formation, S100B activates the PI 3-kinase/AKT pathway and subsequently the NFκB pathway, resulting in cellular proliferation. S100B exerts trophic effects on neurons and astrocytes at lower concentrations and causes neuronal apoptosis, activating astrocytes and microglia at higher concentrations [143-146]. S100B activation of RAGE upregulates IL-1β and TNF-α expression in microglia and stimulates AP-1 transcriptional activity through JNK signaling. Upregulation of COX-2, IL-1β and TNF-α expression in microglia by S100B requires the concurrent activation of NF-κB and AP-1.

#### S100P

S100P binds to RAGE and is important in prostate, pancreas, and gastric cancers [146,147]. It is also detected in normal lung as well as lung cancer tissue, and is increased primarily in adenocarcinomas [148]. Treatment of pancreatic cell lines with S100P stimulates cell proliferation, migration, invasion, and activates the MAP kinase and NFκB pathways [149]. The anti-allergy drug cromolyn binds S100P and will block S100P-RAGE interaction. It inhibits tumor growth and increases the effectiveness of gemcitabine in experimental animal models [150]. Non-Steroidal Anti-Inflammatory Drugs (NSAIDs) are simultaneously pro-tumorigenic by up-regulating S100P expression and anti-tumorigenic by decreasing Cox2 activity [151].

### S100 Proteins – subtle differences translate to large changes in RAGE binding

Although the S100 proteins share much structural similarity with their two EF-hand Ca++ binding domains flanked by α-helices, only some of the members activate RAGE [97]. Subtle structural differences that lead to different biochemical properties (Ca++ and Zn++ binding and preferred oligomerization state) thus seem to lead to different abilities to activate RAGE. Higher oligomerization states tend to lead to RAGE activation. RAGE also binds to several protein families that readily form aggregates and oliogmers – Amyloid beta peptide, Collagen, and AGEs.

### RAGE and Abeta

The Amyloid-beta peptide (Abeta) is a peptide most commonly of 40 or 42 amino acids whose accumulation in amyloid plaques is one of the characteristics of Alzheimer brains. Abeta exists extracellularly either as a monomer, soluble oligomer, or insoluble fibrils and aggregates. Abeta binds to RAGE on neurons and microglial cells [152]. On neurons, Abeta activation of RAGE will generate oxidative stress and activate NF-KB. Abeta activation of microglia will enhance cell proliferation and migration [153,154]. However other receptors might also mediate Abeta toxicity, since RAGE-independent effects also exist [155]. The V and C1 domains of RAGE bind to Abeta oligomers and aggregates (respectively), and blocking these will prevent Abeta-induced neurotoxicity [156]. Exposure of a RAGE-expressing human neuroblastoma cell line (SHSY-5Y) to Abeta oligomers caused massive cell death, while exposure to Abeta fibrils and aggregates caused only minor cell death. Treatment with blocking antibodies specific to RAGE domains was able to protect against Abeta aggregate- or oligomer-inducuded death (but not fibril-induced death).

### RAGE and Collagen

Unlike other non-embryonic tissues, RAGE is highly expressed in healthy lung and its expression decreases in pathological states. RAGE expression in the lung is a differentiation marker of alveolar epithelial type I (AT I) cells, and is localized to the basolateral plasma membrane [20]. RAGE enhances adherence of these cells to collagen-coated surfaces and induces cell spreading [16]. RAGE binds laminin and Collagen I and IV in vitro, but not fibronectin. Thus RAGE plays a role in anchoring AT I cells to the lung basement membrane, which is rich in Collagen IV [20,157,158]. Absence of RAGE expression in (-/-) mice leads to an increase in spontaneous idiopathic pulmonary fibrosis (IPF). Human lung from late-stage IPF patients showed significant down-regulation of RAGE when compared to healthy lung tissue [20].

### AGEs

Advanced glycation endproducts (AGEs) a broad class of non-enzymatic products of reactions between proteins or lipids and aldose sugars [159]. The reaction between the protein and sugar causes its characteristic browning in food products. The western diet in particular is full of AGEs. Although glycation is a general term for addition of a sugar, in this case it specifically refers to non-enzymatic addition to a protein. "Glycosylation" is often used for enzymatic addition of sugars. The Maillard reaction, starting from the glycation of protein and progressing to the formation of AGEs, is implicated in the development of complications of diabetes mellitus, as well as in the pathogenesis of cardiovascular, renal, and neurodegenerative diseases [3,119,160,161]. The Maillard reaction begins with the sugars forming Schiff bases and Amadori products. The carbonyl groups of these precursors can react with amino, sulfhydryl, and guanidinyl functional groups in proteins. AGEs cannot be chemically reverted to their original forms but their precursor, Amadori products, can be. AGEs are a diverse category of non-enzymatic modifications that result for these reactions, and not all AGE-modified proteins activate RAGE. Over twenty different AGE modifications have been characterized, of which carboxymethyl lysine (CML) modified proteins are strong inducers of RAGE signaling [3,160]. Other AGE modifications to proteins (such as pentosidine and pyrraline) do not increase RAGE signaling. As such, characterizing AGE-modifications of proteins is important. One promising technique is Mass Spectrometry, especially "bottom-up" proteomics involving cleavage of proteins followed by analysis of the subsequent peptides [160].

### RAGE and AGEs in the Redox Environment

AGE accumulation itself is considered a source of oxidative stress. In hyperglycemic environments, glucose can undergo auto-oxidation and generate OH radicals [161,162]. Schiff-base products and Amadori products themselves cause ROS production [162]. Nitric Oxide donors can scavenge free radicals and inhibit AGE formation [163]. Over time AGE deposits contribute to diabetic atherosclerosis in blood vessels. As a human naturally ages, one generates high levels of endogenous AGEs [164,165].

RAGE was originally named for its ability to bind AGEs, but since 1995 there have been many more ligands found [8,166]. Formation of AGEs is a way to sustain the signal of a short oxidative burst into a much longer-lived post-translationally modified protein [119]. RAGE will bind to AGE-modified albumin but not nonglycated albumin [167]. AGE activation of RAGE is found in diabetes, neuodegeneration, and aging [168]. Tumors provide an environment that favors generation of AGEs since according to Warburg's original hypothesis they rely primarily on anaerobic glycolosis for energy, and have a higher uptake of glucose [169,170]. Prostate carcinoma cells bind AGEs through the V-domain of RAGE [171]. AGEs have in fact been identified in cancerous tissue, which leads to the possibility of AGE activation of RAGE contributing to cancer growth [172]. However there are also other RAGE ligands in greater abundance. Single molecules of RAGE do not bind AGEs well, but oligomers of RAGE bind them strongly [11]. This supports the notion that RAGE oligomerization is important for sustained signaling. Collagen will normally accumulate some degree of glycation *in vivo*, but collagen with synthetic AGE-modification will enhance neutrophil adhesion and spreading [173].

Sorbinil and zenarestat are orally active aldose reductase inhibitors (ARI's) derived from quinazoline. They, in addition to vitamin C and E, have ameliorative benefits in decreasing intracellular oxidative stress [174]. Vitamin E is effective in part because of its chemical structure. It is able to donate a hydrogen atom from its hydroxyl group, combining with ROS and neutralizing them [175]. Sadly, many of the clinical trials of antioxidants have failed to modify cancer and have in some instances enhanced its development, suggesting that "aerobic" or oxidative extracellular events may be a preferred means to limit chronic inflammation. Injection of soluble RAGE prevents liver reperfusion injury and decreases levels of TNF-α (Tumor Necrosis Factor-α), a cytokine that signals apoptosis and contributes to systemic inflammation, and thereby decreases insulitis [176]. Aminoguanidine delivery also decreases levels of albumin in the blood stream and decreases aortic and serum levels of AGEs thus slowing the progression of atherosclerosis [177].

### RAGE Ligands in Neurobiology

The RAGE-NF-κB axis operates in diabetic neuropathy. This activation was blunted in RAGE (-/-) mice, even 6 months following diabetic induction. Loss of pain perception is reversed in wild type mice treated with exogenous soluble RAGE [178]. The interaction between HMGB1 and RAGE *in vitro *promotes neurite outgrowth of cortical cells, suggesting a potential role of RAGE as a mediator in neuronal development [166]. Nanomolar concentrations of S100B promote cell survival responses such as cell migration and neurite growth. While the interaction of RAGE with S100B can produce anti-apoptotic signals, micro-molar concentrations of S100B will produce oxyradicals, inducing apoptosis. S100B also activates RAGE together with HMGB1, promoting the production of the transcription factor NF-kB [144]. Another proposed mechanism for how RAGE may mediate neurite outgrowth involves sulfoglucuronyl carbohydrate (SGC). Examination of both HMGB1 and SGC in the developing mouse brain reveals that the amount of RAGE expressed in the cerebellum increases with age. Antibodies to HMGB1, RAGE, and SGC inhibit neurite outgrowth, suggesting that RAGE may be involved with the binding of these molecules and their downstream processes [179].

As RAGE may be involved with cell growth and death, its role in cell recovery after injury has also been examined. In rats with permanent middle cerebral artery occlusion, levels of RAGE increase as they do in PC12 cells following oxygen and glucose deprivation (OGD). Blockade of RAGE reduces cytotoxicity caused by OGD [180]. Binding of RAGE to its ligands activates the NF-κB pathway. The presence of RAGE, NF-κB, and NF-κB regulated cytokines in CD4+, CD8+, and CD68+ cells recruited to nerves of patients with vasculitic neuropathies suggests that the RAGE pathway may also play a role in the upregulation of inflammation in this setting [181]. Another RAGE ligand, AGE-CML, is present in endoneurial and epineurial mononuclear cells in chronic inflammatory demyelinating polyneuropathy and vasculitic polyneuropathy [182].

In glioma cells, RAGE is part of a molecular checkpoint that regulates cell invasiveness, growth, and movement. In contrast to lung cancer cells, normal glioma cells express less RAGE than tumor cells. Addition of AGEs to cells stimulates proliferation, growth, and migration. Addition of antibodies targeting RAGE conversely inhibits the growth and proliferation caused by AGEs, increasing survival time and decreasing metastases in immunocompromised mice bearing implanted rat C6 glioma cells [183].

### RAGE in Epithelial Malignancies

The interaction between RAGE and its various ligands plays a considerable role in the development and metastasis of cancer. RAGE impairs the proliferative stimulus of pulmonary and esophageal cancer cells [184]. RAGE is highly expressed in Type-I pneumatocytes, specifically localized in the alveolar epithelium. Interestingly, over-expression of RAGE leads to lower cell proliferation and growth, while downregulation of RAGE promotes development of advanced stage lung tumors [19,185]. Furthermore, blocking AGE-RAGE interactions leads to diminished cell growth [186]. Cells expressing RAGE have diminished activation of the p42/p44-MAPK pathway and growth factor production (including IGF-1) is impaired. RAGE ligands detected in lung tumors include HMGB1, S100A1, and S100P. In pulmonary cancer cells transfected with a signal-deficient form of RAGE lacking the cytoplasmic domain, increased growth when compared to fl-RAGE-transfected cells is noted. Over-expression of RAGE on pulmonary cancer cells does not increase cell migration, while signal deficient RAGE does [187].

### RAGE and Immune Cells

RAGE also acts as an endothelial adhesion receptor that mediates interactions with the β2 integrin Mac-1 [29]. HMGB1 enhances RAGE-Mac1 interactions on inflammatory cells, linking it to inflammatory responses (Table 4) [71,72]. Neutrophils and myelomonocytic cells adhere to immobilized RAGE or RAGE-transfected cells, and this interaction is attributed to Mac-1 interactions [24,71]. RAGE is highly expressed in macrophages, T lymphocytes, and B lymphocytes [188]. RAGE expressed on these cell types contributes to inflammatory mechanisms. The activation of RAGE on T-Cells is one of the early events that leads to the differentiation of Th1+ T-Cells [189]. RAGE is also a counter-receptor for leukocyte integrins, directly contributing to the recruitment of inflammatory cells in vivo and in vitro. Soluble RAGE has been postulated as a direct inhibitor of leukocyte recruitment [190]. RAGE-mediated leukocyte recruitment may be particularly important in conditions associated with higher RAGE expression, such as diabetes mellitus, chronic inflammation, atherosclerosis or cancer [33]. RAGE can directly mediate leukocyte recruitment, acting as an endothelial cell adhesive receptor and attracting leukocytes. RAGE causes an "indirect" increase in inflammatory cell recruitment due to RAGE-mediated cellular activation and upregulation of adhesion molecules and proinflammatory factors [190]. S100A12 and S100B activate endothelial, vascular smooth muscle cells, monocytes and T cells via RAGE, resulting in the generation of cytokines and proinflammatory adhesion molecules [24,67,68].

**Table 4:** Major Immune Cells Expressing or Responding to RAGE-expressing Cells

**Immune cell**	**Associated RAGE ligand**	**Effects on immune cells**	**Associated diseases**
Neutrophils	AGE, Mac-1	Neutrophils adhere to RAGE-transfected cells but free AGE reduces this adherence and the ability of neutrophils to kill phagocytosed microorganisms (bacteria); This adherence elevates intracellular free calcium levels in humans. Upregulation of RAGE was not found after binding.	Diseases where AGE has been implicated (diabetes atherosclerosis, and Alzheimer's disease)

T Cells	HMGB1	RAGE activation is one of the early events in differentiation and proliferation of Th1+ cells	Arthritis

B Cells	HMGB1-CpG DNA	Stimulates cytokine release along with TLR9	Sepsis

Macrophages, Monocytes	Any RAGE ligand	Inflammatory response is generated. Increased conversion of monocytes to macrophages. RAGE activation leads to destruction of macrophages.	Diabetes

Dendritic Cells	HMGB1, some S100's	Antigen presenting capacity is unaffected. RAGE expression is upregulated after cellular activation.	Arthritis

RAGE expression on T cells is required for antigen-activated proliferative responses [189]. RAGE deficient T cells decrease production of IL-2, IFN-γ, and Th1 while producing more IL-4 and IL-5 as Th2 cytokines. RAGE activation thus plays a role in balancing Th1 and Th2 immunity. RAGE deficient dendritic cells appear to mediate rather normal antigen presentation activity and migration both *in vivo *and *in vitro*. RAGE expression is however required by maturing DCs to migrate to draining lymph nodes [191].

## Conclusion

RAGE and its ligands play essential roles in inflammation, neurobiology, cancer, and numerous other conditions. Each ligand distinctly activates RAGE and contributes to the innate and adaptive immune responses as well as modulating, in complex and poorly understood ways, the ability of a variety of cell types to expand and respond to exogenous growth factors. Further studies on RAGE ligands should include focusing on and characterizing changes in signal transduction and inflammatory mechanisms. Other therapeutic molecules besides soluble RAGE may be important to inhibit RAGE activation and, in the setting of cancer, tumorigenesis. RAGE is the link between inflammatory pathways and pathways promoting tumorigenesis and metastasis. Characterizing the role of RAGE *in vivo *and *in vitro *can be broadly applied to a variety of pathological conditions and incorporated into a wide array of treatment regimens for these conditions.

## Competing interests

The authors declare that they have no competing interests.

## Authors' contributions

LJS, DT, RK, DA-A, NA, JI, RR, BL, AAA, HJZ, MTL all 1) have made substantial contributions to analysis and interpretation of published findings; 2) have been involved in drafting the manuscript or revising it critically for important intellectual content; and 3) have given final approval of the version to be published.

## Authors' Information

NA worked at Fox Chase Cancer Center through the Howard Hughes Medical Institute Student Scientist Program and currently attends the University of Pennsylvania where he works in a radiation oncology lab studying the effects of hypoxia on brain tumor cells. BL, JI, and RR all worked along with NA in the AMP Program of the Jack Kent Cooke Foundation and are currently at Harvard University as undergraduate students. Drs. Joan Harvey and Michael T. Lotze [University of Pittsburgh], Matthew Albert [Pasteur, Paris], W. Herve Fridman and Catherine Sautes [Universite Pierre e Marie Curie, Paris], and David Chou [NIAID, Bethesda] served as mentors in this program.

LJS, DT, RK, HJZ, AAA, and MTL are part of a coalition of laboratories known as the DAMP Lab. It was formed in 2006 at University of Pittsburgh to focus on the role of Damage Associated Molecular Pattern Molecules [DAMPs] released or secreted by damaged or injured cells or the inflammatory cells responding to the "danger". Along with Dr. Michael E. de Vera and Dr. Xiaoyan Liang, they focus on the critical role of DAMPs in the initiation of chronic inflammation and the disease that often eventuates as a consequence, cancer.
